# 8.B. Workshop: The Dutch GOR-Covid-19 health monitor: a five-year research program to inform public health policy

**DOI:** 10.1093/eurpub/ckac129.478

**Published:** 2022-10-25

**Authors:** Organised by: National Institute for Public Health and the Environment, GGD GHOR Netherlands, NIVEL, ARQ National Psychotrauma Centre (Netherlands), Chair persons: Michel Dückers (Netherlands), Elske Marra (Netherlands)

## Abstract

Societies can be confronted with disasters and major incidents that form a serious threat to population well-being, safety and health. The Covid-19 pandemic challenged governments at different levels in many countries across the world for over two years to address a variety of public health risks. This also applies to the Netherlands, a densely populated high income country in Europe with approximately 17 million citizens. Experience with previous crises - such as the Bijlmermeer Airplane Disaster in Amsterdam (1992), the Q fever outbreak (2007-2011), the MH17 airplane disaster in Ukraine (2014) and gas-mining induced earthquake problems in the north of the Netherlands (ongoing) - shaped the current Dutch public health response structure and knowledge base, designed to assist local and national authorities to anticipate disaster-related population health risks. During the Covid-19 pandemic this structure was used as a starting point to develop and implement a five-year monitoring program: the integrated GOR-Covid-19 health monitor; GOR stands for “Gezondheidsonderzoek bij rampen” (disaster health research). The main objective of the GOR-Covid-19 monitor is to collect and promote the uptake of reliable and timely monitoring information for the benefit of regional and national public health decision-making in the Netherlands, focusing on the public health impact of the Covid-19 crisis (virus infections and mitigation measures) among children and adolescents, adults and public mental health care, a potentially vulnerable group including homeless and former drug addicts. The monitor is carried out under coordination of the “Network GOR”, comprised of the National Institute for Public Health and the Environment, GGD GHOR Netherlands (on behalf of the 25 municipal health regions), Nivel, and ARQ National Psychotrauma Centre. The current workshop consists of five presentations by members of Network GOR. The first contribution gives an overview of the research objectives and the outline of the study protocol of the monitoring program. The other contributions focus on particular study components, that are shaped and aligned during the timeline of the monitor. One presenter will share the findings from the first series of systematic literature reviews. Two contributions are based on a combination of surveys in representative samples and an analysis of primary care registry data: one with an emphasis on quarterly results (short-cycle monitoring), the other on (bi-)annual reports, which zoom in on long-term developments and differences between subgroups based on vulnerability factors and geography (long-cycle monitoring). The fifth contribution details the participatory stakeholder-engagement process designed and implemented to promote that monitor findings are used for local and national public health sense-making and decision-making.

**Key messages:**

• The workshop describes the background and design of a national longitudinal monitoring program to track the public health impact of the Covid-19 pandemic in the Netherlands.

• An overview is given of the first findings on the health impact of the pandemic for different populations and related vulnerability factors as well as the stakeholder engagement process.

